# Pattern of injuries amongst tennis players in Accra, Ghana

**DOI:** 10.4102/sajp.v76i1.1429

**Published:** 2020-07-22

**Authors:** Gabriella Acquaye, Jonathan Quartey, Samuel Kwakye

**Affiliations:** 1 Physiotherapy Department, Holy Trinity Hospital, Accra, Ghana; 2 Department of Physiotherapy, School of Biomedical and Allied Health Sciences, University of Ghana, Accra, Ghana; 3 Physiotherapy Unit, Medical Department, West African Football Academy, Sogakope, Ghana

**Keywords:** tennis, injury prevention, tennis player, pattern of injury, tennis injury, overuse injury

## Abstract

**Background:**

Tennis is a popular global sport characterised by repeated, explosive motions and the involvement of several muscle groups during different strokes, which fluctuates randomly from brief periods of maximal or near maximal work to longer periods of moderate and low intensity activity.

**Objectives:**

To determine the pattern of injuries amongst tennis players in Accra.

**Method:**

A cross-sectional study was conducted, involving 142 male and female participants selected from tennis clubs in Accra and the Accra sports stadium. A standardised tennis injury report form was used to obtain data from participants. Data on the parts of the body mostly injured and the types of injury mostly sustained by the players were summarised and presented appropriately with the use of graphs and pie charts. Assessment of the causes underlying the prevailing injuries to tennis players was also tabulated and comparisons made.

**Results:**

Out of a total of 170 injuries recorded, knee (39 [27.5%]) and shoulder (31 [21.1%]) injuries were the most commonly sustained. Most (80 [56.3%]) tennis injuries occurred during training. Other injuries (26 [18.3%]) occurred during competitions or tournaments whilst 26 (18.3%) occurred during social play. About 10 (7.0%) participants were not certain of the type of activity at the time of injury. The majority (35 [24.65%]) of the players received no treatment for their injuries. However, few of the injuries (20 [14.08%], 14 [9.86%], 6 [4.23%]) sustained were treated by medical personnel, physiotherapists or nurses respectively. There was no association between warm-up before play and cause of injury (*p* = 0.375). There was also no association between shoe type and cause of injury (*p* = 0.253).

**Conclusion:**

The majority of the injuries occurred in the upper and lower limbs. Most of these injuries occurred during training with overuse and overexertion being the most common cause.

**Clinical implications:**

It is important to educate tennis players and coaches on injury prevention measures and the use of protective gear during tennis.

## Introduction

Tennis is a popular sport globally with participants from different age groups participating in more than 200 countries affiliated with the International Tennis Federation (Kachanathu, Kumar & Malhotra 2014). Tennis is characterised by repeated, explosive motions and the involvement of several muscle groups during different strokes, which fluctuate randomly from brief periods of maximal or near maximal work to longer periods of moderate and low intensity activity (Arora & Doshi 2014). Unlike many sports, the period of play for tennis matches is not time bound and, thus, matches can last for several hours (Abrams, Renstrom & Safran 2012). This long period of play combined with the variety of strokes can result in a unique profile of injuries (Chevinsky et al. 2017; Dines et al. 2015). The volume of play combined with the physical demands of the sport imposes a high load of strain which predisposes the tennis players to injuries that result from a number of inter-related intrinsic and extrinsic factors (Arora & Doshi 2014). It also places acute physical demands on players as the game relies on repetitive stopping, starting, pivoting, twisting, and flexion motions throughout the game whilst maintaining sufficient balance, control and upper body strength to hit the ball effectively (Colvin & Andelman 2016).

There are a number of factors that can increase a player’s risk of an injury. These include overtraining, inadequate muscular strength or endurance, inflexibility, improper equipment, previous history of injury and a poor aerobic fitness level (Hjelm, Werner & Renstrom 2012). The weight of the racquet can also contribute to wrist injuries if not suitable to the tennis player (Martin et al. 2014). Player-specific factors, such as age, sex, volume of play, skill level, racquet properties and grip positions as well as the playing surface are some of the risk factors that can predispose a tennis player to injury (Kachanathu et al. 2014). Muscle strain is one of the most common injuries in tennis (Kazemi, Rahnama & Alizadeh 2016). Overuse of a muscle is also a common cause of injury in tennis players and can result in muscle, cartilage, nerve, bursae, ligament and tendon damage (Hjelm et al. 2012). For instance, Pluim et al. (2015) reported that the average weekly prevalence of all health problems was 21.3% of which 12.1% constituted overuse injuries amongst elite tennis players. The repetitive use of a particular muscle resulting in a micro-tear without time for repair and recovery is the most common cause of injury (Levangie & Norkine 2011). Most (37.9%) injuries amongst tennis players are sustained by a nonspecific mechanism during play (Gaw, Chounthirat & Smith 2014). Most (83.4%) of these injuries occur at a sport or recreation facility with children aged 5 to 18 years having a higher mean injury rate than adults older than 19 years (Gaw et al. 2014). The most commonly injured body regions according to Gaw et al. (2014), were the lower extremities (42.2%) and upper extremities (26.7%). In contrast, Maquirriain and Baglione (2016) reported that the upper limbs were the most commonly injured body part amongst tennis players. The use of correct sports outfits, such as appropriate shoes for play, tend to reduce the impacts of the courts’ surfaces on the knee and hence reduce the incidence of injuries to the knee (Asker et al. 2018).

According to Kachanathu et al. (2014), an overall injury incidence of 2.18 injuries per 1000 tennis playing hours was recorded during tennis training sessions and tournaments, and the prevalence of injury was found to be 15.62 injuries per 100 players. Kachanathu et al. further suggest that education of tennis players on tennis injuries should be reinforced to reduce the occurrence of injuries.

Injuries are one of the limiting factors that could prevent the success of a tennis player in a competition or training sessions (Fu et al. 2018). In developed countries, there seems to be an ascendency of documentation of injuries for various sporting disciplines, including tennis, which has helped in developing rehabilitative and preventive measures to reduce sports related injuries in those disciplines (Abrams et al. 2012; Gaw et al. 2014; Plium et al. 2016).

In spite of the reports indicated about tennis high income countries, it appears there is a paucity of similar studies in Ghana at elite, professional and recreational levels for tennis players. This invariably makes it difficult to develop proper injury prevention strategies to help minimise or prevent the occurrence of such injuries amongst the players, which will help them achieve maximum performance. Hence, the aim of our study was to initially determine the pattern of injuries amongst tennis players in Accra.

### Methods

This cross-sectional survey was conducted at four selected tennis clubs in Accra as well as at the tennis courts of the Accra sports stadium which is recognised by the Ghana Tennis Federation amongst all other clubs in Ghana. They were Armstek, Zoti, Atomic and Accra Lawn Tennis Clubs. The clubs were selected because members were consistent with training, social play and competitions and they are recognised by the Ghana Tennis Federation as such. The study involved male and female players between the ages of 18 and 60 years who play tennis at least three times per week. Male and female players who participated in other sport disciplines apart from tennis were excluded. The sample size obtained was 138 using the unknown sample size formula: *n* = *P* (1−*P*) (Z/E)^2^.

A standardised tennis injury report form (Appendix 1) designed by Sports Medicine Australia (SMA) was used to obtain data. An additional data capturing form (Appendix 2) designed by the first author was also used to obtain information from participants.

The tennis injury report form had 13 sections which sought information on: the type of activity at the time of injury, reason for the presentation of injury, body parts injured, nature of the injury, mechanism or cause of injury, how the injury occurred, participants’ perceived reasons for the cause of the injury, protective equipment if worn at all during play, type of treatment received for the injury, action participants take to return to play, information on referral, severity of injury and person who treated.

The data capturing form (Appendix 2) was used to retrieve information on the age and gender of the participant, racquet grip positions and properties, volume of play, skill level of the tennis player, warm-up duration, effect of injury during tennis training, competitions or social play, the number of years of tennis experience and the number of days played in a week.

An introductory letter which clearly stated the purpose of the study from the Head of the Physiotherapy Department, University of Ghana, was delivered to the President of the Ghana Tennis Federation and head coaches of the selected clubs, after ethics approval was sought and obtained.

The authors interacted with the head coaches and tennis players before and after tennis matches, social play or training at the play courts of selected clubs. The purpose of our study was explained after which participants were asked to complete a written consent form. Participants were also assured of anonymity and confidentiality.

A detailed explanation of the tennis injury report and data capturing forms was provided to participants who agreed to take part in our study.

The authors interacted with participants to inquire about any injury sustained as a result of playing tennis. If a player had sustained an injury as a result of playing tennis, the player qualified to participate in our study. Injuries were physically assessed by the authors to confirm the type of injury sustained. Each participant was assessed once by the authors. The participants subsequently completed the data capturing form. The tennis injury report and data capturing forms took about 5 min to complete. Participants who couldn’t take part in the study as a result of time constraints were asked to complete the data capturing form at their convenience and these were retrieved on subsequent visits to the courts, within 2 weeks. The authors assessed the injuries of those participants upon returning the data capturing form. Data collection lasted 8 weeks (March and April of 2018).

The data were coded and analysed using SPSS version 23.0. Descriptive data with summary statistics of mean and standard deviation were computed for the observed variables and presented in graphs, tables and charts. Data, on the parts of the body mostly injured and the types of injury mostly sustained by the players, were summarised and presented appropriately with the use of graphs and a pie chart. The responses helped describe the proportions of injury types and body parts injured. Assessment of the causes underlying the prevailing injuries to tennis players was also tabulated and comparisons were made.

### Ethical consideration

Ethical clearance was sought and obtained from the Ethics and Protocol Review Committee, School of Biomedical and Allied Health Sciences, University of Ghana reference number: SBAHS-PH/10517563/SA/2017-2018.

## Results

A total of 142 participants took part in our study. The youngest participant was 18 and the oldest was 59 years with an average age of 37.52 ± 11.82 years. The average number of days played in a week was 4.04 ± 1.7 and the average years of tennis playing experience was 15.09 ± 11.88. The majority (132 [93.0%]) of the participants were right hand dominant. Table 1 provides details of the demographics and characteristics of participants. The majority (61 [43.0%]) of participants’ injuries were moderately severe followed by severe injuries (47 [33.1%]) with a few (34 [23.9%]) mild injuries.

**TABLE 1:** Demographics and characteristics (*n* = 142).

Variable	Frequency	%
**Sex**		
Male	119	83.8
Female	23	16.2
**Hand grip dominance**		
Right	132	93.0
Left	10	7.0
**Diameter of racquet**		
Small	21	14.8
Medium	76	53.5
Large	45	31.7
**Racquet grip**		
Eastern	33	23.2
Semi-western	19	13.4
Continental	62	43.7
Western	28	19.7

The site of most commonly sustained injuries was the knee (39 [27.5%]) followed closely by the shoulder (31 [21.1%]). The majority of the players’ injuries occurred in the upper limbs (89 [52.4%]). Details of the pattern of injuries amongst the players are presented in Table 2.

**TABLE 2:** Pattern of injuries amongst the players (*n* = 142).

Injury	Frequency	%
**Upper limb**	89	52.35
Shoulder	31	18.24
Arm	5	2.94
Elbow	26	15.29
Wrist/hand	27	15.88
**Lower limb**	76	44.71
Hip/thigh	17	10
Knee	39	22.94
Ankle	20	11.76
**Others**	5	2.94
Neck	1	0.59
Back	1	0.59
Spine	3	1.76
Total injuries	170	100

Most (80 [56.3%]) participants reported that tennis injuries occurred during training whilst 26 (18.3%) indicated that they occurred during competitions or tournaments and 36 (25.3%) reported that they occurred during social plays.

Ten (7%) weren’t certain of the type of activity at the time of injury. The commonest reason for referral (58 [40.8%]) was injury aggravation. New injuries or acute injuries numbered 51 (35.9%) whilst 35 (24.6%) of the injured participants were wearing protective equipment at the time the injury occurred during competition, training and social play as shown in Table 3.

**TABLE 3:** Reason for referral and use of protective equipment (*n* = 142).

Reason for referral	Frequency	%
New injury	51	35.9
Aggravated injury (symptoms worsened after tennis play)	58	40.8
Recurrent (symptoms occur without any known factor)	27	19.0
Others	5	3.5
**Was protective equipment worn on the injured site?**		
Yes	35	24.6
No	107	75.4

**FIGURE 1:**
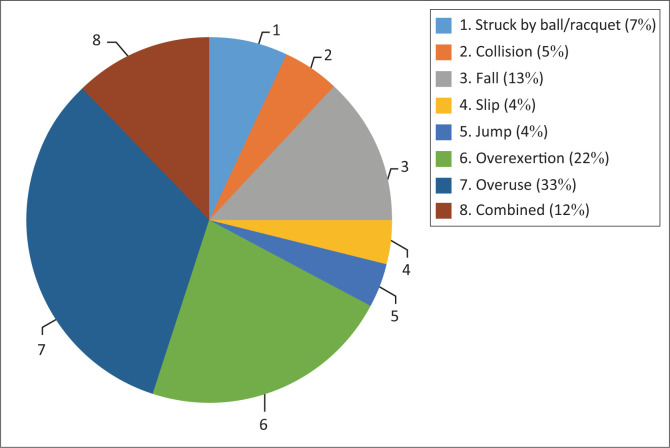
Mechanism of injury.

Overuse and overexertion were the most common causes of injury amongst the tennis players, whereas players being struck by the racket or tennis balls were the least cause of injury. Figure 1 provides details about the mechanisms of injury. The majority (35 [24.65%]) of the players did not receive any treatment for injuries sustained.

**FIGURE 2:**
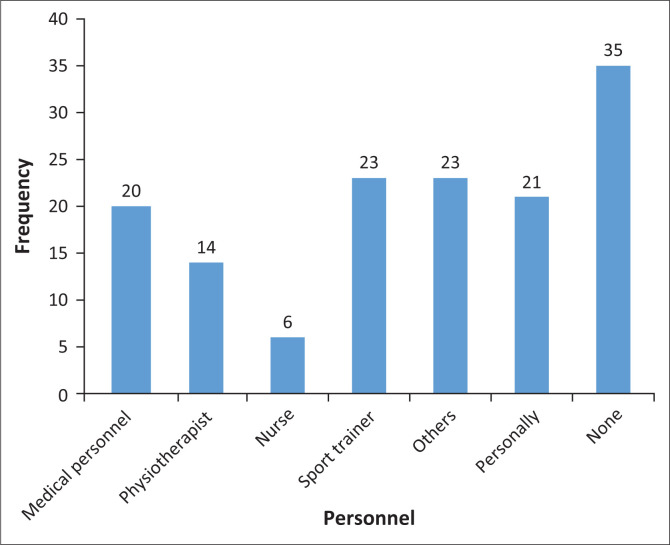
Personnel managing injury.

Twenty (14.08%), fourteen (9.86%) and six (4.23%) of the injuries sustained were treated by medical personnel, physiotherapists and nurses respectively. Figure 2 indicates the available personnel and frequency of usage.

**FIGURE 3:**
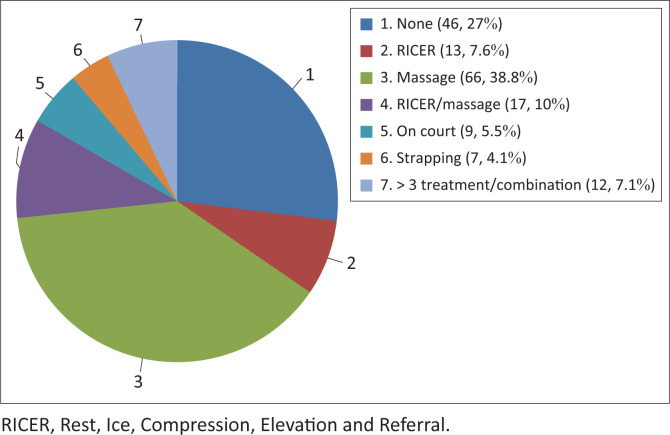
Initial treatment given.

A little above half (78 [54.9%]) of the tennis players warm up before training and 105 (73.9%) were in the intermediate category. The majority (113 [79.6%]) of the tennis players play on a hard surface court whilst most (120 [84.5%]) of them use tennis shoes for playing. Table 4 highlights some possible risk factors that could lead to injuries amongst tennis players. The major (66 [38.8%]) form of treatment received was massage and the least (7 [4.1%]) treatment method was strapping the injured site. Figure 3 depicts details of the initial treatments received by the injured participants. The majority (64 [38%]) of the players’ injuries were strains whilst only five (3%) were abrasions and bruises.

**TABLE 4:** Level of skill, warm-up, court surface and shoe type (*n* = 142).

Variable	Frequency	%
**Warm-up**		
Yes	78	54.9
No	25	17.6
Sometimes	39	27.6
**Level of skill**		
Beginner	5	3.5
Intermediate	105	73.9
Professional	32	22.5
**Court surface**		
Hard	113	79.6
Clay	7	4.9
Grass	2	1.4
Carpet	1	0.7
More than one surface	19	-
**Shoe type**		
Tennis shoe	120	84.5
Cleated	4	2.8
Running shoe	14	9.9
Walking shoe	1	0.7
More than one shoe type	3	2.1

One hundred and forty-two responses were evaluated concerning whether or not warm-up before play had an effect on the cause of injury at a confidence interval of 95%. There was no association between warm-up before play and cause of injury (*p* = 0.375) as well as between shoe type and cause of injury (*p* = 0.253). Table 5 shows the association between warm-up before play and cause of injury and Table 6 shows the association between shoe type and cause of injury.

**TABLE 5:** Association between warm-up before play and cause of injury.

Test	Value	df	Asym. sig. (2-sided)
Pearson chi square	10.779†	10	0.375
Likelihood ratio	14.940	10	0.134
*N* of valid cases	142	-	-

df, degrees of freedom.

**TABLE 6:** Association between shoe type and cause of injury.

Test	Value	df	Asym. sig (2-sided)
Pearson chi square	56.244†	50	0.253
Likelihood ratio	38.213	50	0.888
*N* of valid cases	142	-	-

df, degrees of freedom.

## Discussion

The majority of the tennis players who were available for recruitment were male. This could be attributed to the fact that tennis is played by more men than women especially because it is a game characterised by explosive motions requiring power and speed.

Overuse injuries were the most common causes of injuries recorded amongst the tennis players which corroborates reports by Hjelm et al. (2012) and Fu et al. (2018). In contrast, Kazemi et al. (2016) reported muscle twist as the most common mechanism of injury. However, muscle twist or fall was the dominant cause of injury after overuse and over exertion amongst participants. Some preventive measures for overuse injuries amongst tennis players include the use of the correct sports outfits, such as appropriate shoes for play, to reduce the impact of the court’s surface on the knee, as well as using dampeners (a small device usually made of rubber that is inserted into a tennis racquet string bed) to prevent prolonged vibration of the racquet on the wrist (Asker et al. 2018).

Our findings showed that most injuries occurred in the upper limbs, contrary to findings by Maquirriain and Baglione (2016), who reported that the incidence of lower-limb injuries amongst tennis players was higher than upper-limb and trunk lesions. Although most injuries occurred in the upper limbs, the knee was the most frequently injured body part, followed by the shoulder which is similar to findings by Hjelm et al. (2012). This may be a result of the sudden changes made by the tennis players in order to reach the ball during tennis play (Dines et al. 2015).

Although Abrams et al. (2012) reported that tennis places high loads on the knee, with repetitive short movements from side to side causing injuries to the lower extremity, some other studies (Chevinsky et al. 2017; Dines et al. 2015) report that the ankle was the most commonly injured body part amongst tennis players. This outcome could be attributed to side to side shuffling which may lead to ankle twist, fall or slide resulting in ankle injuries (Gaw et al. 2014).

The shoulder was the second most injured body part amongst the participants. This outcome can be attributed to the service motion which is considered the most difficult and powerful stroke in tennis and could, over time, result in shoulder injuries, as reported by Abrams et al. (2012).

Martin et al. (2014) indicate that a large force is generated in the upper limb in order to be able to hit a ball played by an opponent with power, and this could cause transmission of vibrations through the racquet to the shoulder causing an imbalance in the kinetic chain which may lead to shoulder injuries. Injuries to the wrist can be attributed to the vibrations from the tennis racquet. The weight of the racquet could contribute to wrist injuries if not suitable to the tennis player, for instance if the racquet is too heavy for the tennis player to use (Martin et al. 2014). The tennis player usually uses more force in keeping the grip of the racquet firm as a result of the relatively heavy weight of the racquet and might cause injuries to the wrist when receiving powerful shots from an opponent.

A high number of the tennis players did not receive appropriate treatment as a result of inadequate knowledge about correct treatment and the availability of services offered by a sports medical team. However, one a season prospective study of injuries and illness in elite junior tennis players in the Netherlands reported a decrease in tennis injuries because of the availability of appropriate medical services (Pluim et al. 2015). For this same reason, most injured tennis players find their own means of treatment, which is usually massage to the injured area.

Show that forty six percent of participants did not receive any form of treatment. Very few injured tennis players received treatment from professional members of the sports medical team. This may be because most injured tennis players did not know the significance of receiving the right treatment from the right professionals. Physiotherapists and other medical personnel who are part of the sports medical team were mostly not readily available to treat injured tennis players.

Tennis players should be educated about the game in general, with an emphasis on correct techniques to be used during play. Preventive measures such as using the correct protective equipment (a dampener to reduce vibrations in the racquet, wearing the correct footwear appropriate for tennis and the type of court being played on, amongst others) should be in place to reduce the occurrence of injury. Physiotherapists should also prescribe appropriate strengthening exercises for the upper and lower limbs which could also serve as preventive measures for the high occurrence of shoulder and knee injuries amongst tennis players. The Ghana Tennis Federation could also make it a policy for health personnel, such as physiotherapists, to be employed by the various clubs, school teams and tennis facilities for the proper management of injuries during training sessions, competitions and social games.

## Conclusion

The results provide a useful insight into the mechanisms, sites, nature, severity, possible risk factors and possible managements of injuries amongst tennis players in Accra. The majority of the injuries occurred in the knee and shoulder. Most of the injuries were caused by overuse and or overexertion often during training sessions. Most injuries were not treated immediately as expected, as a result of a lack of knowledge on the correct treatment and the availability of appropriate medical services.
